# Unveiling the Synergistic Effect of Ferroelectric Polarization and Domain Configuration for Reversible Zinc Metal Anodes

**DOI:** 10.1002/advs.202105980

**Published:** 2022-03-10

**Authors:** Tao Chen, Fei Huang, Yinan Wang, Yi Yang, Hao Tian, Jun Min Xue

**Affiliations:** ^1^ School of Chemistry and Chemical Engineering Nanjing University of Science and Technology Nanjing 210094 China; ^2^ Department of Materials Science and Engineering National University of Singapore Singapore 117575 Singapore; ^3^ School of Physics Harbin Institute of Technology Harbin 150001 China; ^4^ School of Mathematical Science Peking University Beijing 100871 China

**Keywords:** aqueous Zn‐ion batteries, domain configuration, ferroelectric polarization, ferroelectric/electrode interface, Zn metal anode

## Abstract

The tendency of zinc (Zn) anodes to form uncontrolled Zn electrodeposits and the occurrence of side‐reactions at Zn‐electrolyte interfaces are a fundamental barrier hampering broad applications of aqueous rechargeable Zn‐based batteries. Herein, a ferroelectric domain‐mediated strategy is proposed to manipulate the Zn plating behavior and achieve controllable Zn growth orientation by coating Zn foil with a ferroelectric tetragonal KTN (t‐KTN) layer. The ferroelectric domain of t‐KTN single crystals exhibits periodic distribution of upward and downward polarizations, corresponding to alternating positively and negatively charged surfaces. The charged ferroelectric surfaces can manipulate the transfer kinetics of Zn ions and the concentration distribution of anions via the interplay between ferroelectric dipoles and adsorbed ions. With the synergistic effect of the ferroelectric polarization and domain configurations, the well‐aligned interlamellar arrays composed of electrodeposited Zn are formed in the initial deposition process, which enable selective deposition within interlamellar arrays and eliminate the dendrite growth during the following plating process. As a result, the t‐KTN layer‐modified Zn anode enables reversible Zn plating/stripping with low voltage hysteresis for over 1200 h at 1 mA cm^−2^ in symmetric cells, and the assembled full cell exhibits a significantly enhanced cycling stability of over 5500 cycles at 5 A g^−1^.

## Graphical abstract

A ferroelectric domain‐mediated strategy is proposed to manipulate the zinc (Zn) plating behavior and achieve controllable Zn growth orientation by a ferroelectric tetragonal KTa_0.54_Nb_0.46_O_3_ (t‐KTN) coating. With the synergistic effect of the ferroelectric polarization and domain configurations, there forms well‐aligned Zn interlamellar arrays with low tortuosity in the initial stage of Zn deposition, enabling selective deposition within interlamellar arrays.

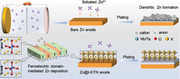

## 1 Introduction

Recently, emerging aqueous Zn‐ion batteries have been viewed as a compelling complement to conventional lithium‐ion batteries for large‐scale energy storage systems because of their environmental benignity, operational safety, high abundance, and low cost.^[^ ^1^, ^2^, ^3^ ^]^ Metallic Zn with a high volumetric capacity (5855 mAh cm^−3^) and a moderate redox potential (−0.76 V vs the standard hydrogen electrode) is the most common anode material for aqueous Zn metal batteries (AZMBs).^[^ ^4^, ^5^, ^6^, ^7^, ^8^ ^]^ Unfortunately, the commercialization of rechargeable AZMBs is severely impeded by the notorious growth of Zn dendrites, which results in low Coulombic efficiency (CE), fast capacity fading, and poor cycle life of batteries.^[^ ^9^, ^10^, ^11^, ^12^ ^]^ During initial Zn plating, the intrinsic surface roughness of Zn foil would give rise to an uneven electric field distribution.^[^ ^13^, ^14^ ^]^ Accordingly, Zn ions prefer to accumulate and nucleate at the tips of the protuberances with enhanced electric field, leading to uneven distribution of Zn ion concentration and local current densities.^[^ ^15^, ^16^ ^]^ Consequently, Zn dendrites continuously grow and eventually penetrate the separators, causing internal short circuits and the battery failure.^[^ ^17^ ^]^

Extensive efforts have been dedicated to tackling the issue of Zn dendrites, including interfacial engineering by building artificial protective layers,^[^ ^18^, ^19^, ^20^, ^21^, ^22^ ^]^ optimizing electrolyte components,^[^ ^23^, ^24^, ^25^, ^26^, ^27^ ^]^ designing 3D host structures,^[^ ^28^, ^29^, ^30^ ^]^ modifying the separators,^[^ ^31^, ^32^, ^33^ ^]^ and controlling the preferential orientation of Zn deposition.^[^ ^5^, ^14^, ^34^, ^35^, ^36^, ^37^ ^]^ Among all these strategies, the construction of an advanced protective layer on Zn anode surface is a highly scalable and low‐cost approach to stabilize Zn metal anode and improve the reversibility of Zn plating/stripping. Although these protective layers can suppress dendrite growth to a certain extent, Zn dendrites are still inherently unavoidable under harsh conditions such as high current densities and deposition capacities.^[^ ^32^ ^]^ This is mainly because dendrite formation process is thermodynamically and kinetically favorable.^[^ ^38^, ^39^ ^]^ Besides, the electric field and ion distribution are two kinetic parameters that have an effect on initial dendrite nucleation and growth during Zn plating process.^[^ ^40^, ^41^ ^]^ On the one hand, the uneven distribution of electric fields around anode surface or freshly plated metal electrodeposits may trigger inhomogeneous or selective nucleation/growth. On the other hand, the Zn ion concentration on anode surface will decrease continuously during the Zn plating process, and the Zn‐ion transport rate is much lower than the Zn deposition rate at the reaction interface. As a consequence, the mismatch between Zn ion transport and deposition rates generally induces Zn ion concentration gradient at the Zn/electrolyte interface and eventually aggravates uneven Zn deposition distribution.^[^ ^12^ ^]^ In view of that, it is highly desirable to develop an effective strategy that could homogenize electric field distribution and accelerate ion transport at the interface, thus achieving highly reversible and long‐life Zn metal anodes.

Recently, ferroelectric materials, such as BaTiO_3_ and *β*‐phase poly(vinylidene fluoride), have already been employed to regulate Li/Zn metal deposition as artificial protective layers.^[^ ^42^, ^43^, ^44^ ^]^ The local uniform orientation of ferroelectric polarization forms as domain, which could be switched by applied electric field. Meanwhile, the polarization can be enhanced by the applied electric field. During the polarization changing process, the ferroelectricity can dynamically accelerate ion transport and homogenize ion flux by creating an internal electric field, thus achieving uniform and lateral deposition. However, preferential accumulation of metal occurs on the upper surface of the electrodes or horizontally‐oriented Zn electrodeposits may block ion‐transport pathway toward the inner electrode.^[^ ^45^ ^]^ Over time, the drastic volume change induced by the accumulated metal plating/stripping could cause poor adhesion between electrode and ferroelectric coatings. So far, there is a lack of deep understanding of the role of domain structure/ferroelectric polarization directions in regulating cation/anion migration and controlling Zn deposition morphology. Therefore, exploring and manipulating domain configurations are very necessary to maximize the advantages of ferroelectric materials for stabilizing Zn metal electrodeposition and constructing high‐performance rechargeable AZMB.

Herein, we report a ferroelectric domain‐mediated strategy to manipulate the Zn plating behavior and achieve controllable Zn growth orientation by a tetragonal KTa_0.54_Nb_0.46_O_3_ (t‐KTN) layer‐modified Zn foil. The ferroelectric‐paraelectric phase transition temperature (Curie temperature, *T*_C_) of KTN can be controlled by adjusting the Ta/Nb composition. In addition, composition gradient of KTN single crystal can affect the orientation of polarization during the phase transition process (from isotropic to anisotropic along crystallographic orientation), aiming at improving the influence of domain configuration on the electric field distribution and ion transport at the reaction interface. In ferroelectric tetragonal KTN crystal, Nb^5+^ shows off‐center displacement along crystallographic orientation inducing spontaneous polarization. Because of the character of spontaneous polarization, the negatively charged ferroelectric surface opposite of polarization direction would attract a large number of Zn ions during Zn plating, thus ensuring a high concentration of Zn ions for the electrodeposition of Zn at the reaction interface. Conversely, the adjacent positively charged surface (along polarization direction) would repel the identically charged cations but attract the oppositely charged anions, thereby avoiding the space charge induced by anion concentration gradient. Benefiting from the synergistic effect of the ferroelectric polarization and domain configurations, the resultant well‐aligned Zn lamellar arrays on the surface of Zn anode can facilitate Zn ion transport and selective Zn deposition within interlamellar arrays. Such a unique deposition behavior can effectively eliminate the formation and growth of Zn dendrites. As a result, the assembled Zn symmetric cells achieve an ultralong cycling life of 1200 h at a current density of 1 mA cm^−2^ and an areal capacity of 1 mAh cm^−2^. Moreover, the full cells using modified Zn anode and vanadium‐based cathode also exhibit excellent rate capabilities and long‐term cycling life.

## 2 Results and Discussion

The ferroelectric t‐KTN single crystals are synthesized by an improved top‐seeded solid growth method, according to the previous report.^[^ ^46^ ^]^ The detailed synthesis process is shown in the Experimental Section. The dielectric permittivity versus temperature curves (Figure S1, Supporting Information) show that the *T*_C_ and orthorhombic–tetragonal phase transition temperature (*T*_O‐T_) of the KTN crystal are 68 and −2 °C, respectively. In other words, composite ferroelectric domains of as‐prepared t‐KTN single crystals are formed at room temperature. The t‐KTN single crystals exhibit a typical solid‐solution perovskite structure. To reveal the origin of internal field in t‐KTN, frequency and temperature dependence *P‐E* loop is shown in Figure S2, Supporting Information. The *P–E* hysteresis loop of t‐KTN single crystals exhibits superior ferroelectricity at room temperature, suggesting the positive and negative saturated polarizations of 18.1 and −18.3 µC cm^−2^. Such high spontaneous polarization feature in perovskite ferroelectric t‐KTN is expected to release and adsorb the free ions/charges at the interface via the interplay/coupling effect between ferroelectric dipoles and adsorbed ions/charges. The coercive field of t‐KTN is approximately 5 kV cm^−1^, which is much higher than the applied electric field of battery. This result indicates that the ferroelectric domain configuration will not be switchable by discharge/charge voltages of aqueous batteries. The ferroelectric domain in the as‐grown t‐KTN crystal was observed using polarizing light microscopy (PLM). As one relaxor‐like ferroelectric oxide, t‐KTN single‐crystal possesses a composite ferroelectric domain structure, showing an obvious 90° domain structure with different polarization directions (Figure S3, Supporting Information).

**Figure 1:**
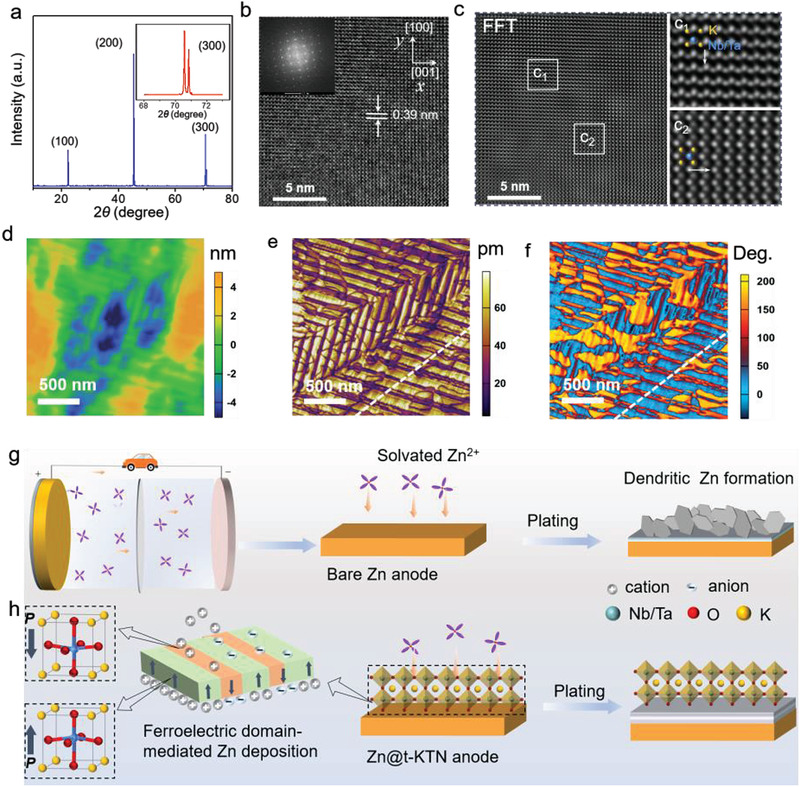
Ferroelectric domain structure of t‐KTN surface and schematic illustration of ferroelectric domain‐mediated strategy for reversible Zn deposition. a) XRD pattern of the t‐KTN single crystal. b) High‐resolution TEM image and selected area electron diffraction (SAED) in inset, corresponding image c) after Fourier transform showing the off‐center displacement of the B‐site ions at the atomic scale in KTN. d–f) The out‐of‐plane piezo‐response force microscope images of 180° domain regions in t‐KTN crystal for (d) surface topography, (e) amplitude, and (f) PFM phase. g) Schematic illustrations of the Zn dendrite growth observed in bare Zn metal anode, and h) ferroelectric domain‐mediated Zn deposition suppressing the dendrite growth via spontaneous upward and downward polarization of t‐KTN, regulating Zn ion distribution at the interface of ferroelectric and Zn electrode.

The phase and structure of the as‐prepared t‐KTN crystals were examined by X‐ray diffraction (XRD). The XRD pattern of t‐KTN shows three obvious diffraction peaks corresponding to the (100), (200), and (300) planes (**Figure** **1**a), indicating these KTN crystals are highly crystallized in the tetragonal phase. The morphology and microstructure of t‐KTN crystals were analyzed by scanning electron microscopy (SEM) and transmission electron microscopy (TEM). SEM image of t‐KTN single crystal (Figure S4a, Supporting Information) displays an irregular shape with a size of ≈150 µm. Moreover, the energy dispersive spectroscopy (EDS) elemental mapping clearly confirms the homogeneous distribution of K, Ta, Nb, and O elements in the t‐KTN crystals without impure elements (Figure S4b, Supporting Information). EDS analysis reveals that the atomic ratio of Ta/Nb is 0.54:0.46, indicating a chemical formula of KTa_0.54_Nb_0.46_O_3_ (Figure S4c, Supporting Information). The high‐resolution TEM image in Figure 1b shows that the interplanar spacing of 0.39 nm is assigned to the (200) lattice plane of t‐KTN crystal. Figure 1c shows an atomic‐resolution scanning transmission electron microscopy (STEM) image of the t‐KTN crystal. The fast Fourier transform of TEM image displays the atomic positions of Nb/Ta and K in polar regions (Figure 1c). The off‐center displacement of the Nb/Ta atoms along the [$\bar{1}$00] and [00$\bar{1}$] directions can be clearly observed, representing as in‐plane 90° domain structure.

To further investigate the ferroelectric domain structures and polarization states, the vertical piezo‐response force microscopy (V‐PFM) was performed at room temperature. The working mechanism is based on the polarization character of the sample under an applied excitation potential using a conducting probe. The domain structure observed by the piezo‐response force microscopy shows a good consistency with the above PLM results. The height retrace image in Figure 1d shows that the surface roughness of t‐KTN crystal is about ±2 nm, indicating that the response signals of domains structure are not related to the surface topography. A sharp contrast in the vertical piezoelectric response amplitude and phase images indicates high piezoelectricity in t‐KTN crystals. Moreover, the t‐KTN crystal exhibits complex domain structures, including strip‐type 180° domains and strip‐type 90° domains (Figure 1e,f and Figures S5 and S6, Supporting Information). The ferroelectric domains of t‐KTN crystal exhibit a periodic distribution of upward and downward polarizations across the domain array, leading to alternating positive and negative bound charges. From the piezo‐responsive phase curve (Figure S7, Supporting Information), the phase angle was changed by 180° under a direct current (DC) bias field, indicating the ferroelectric polarization switching process of t‐KTN single crystals. The amplitude curve shows a well‐defined butterfly hysteresis loop, further revealing the excellent piezoelectric response of t‐KTN crystals.

Zn metal is a hostless electrode that is dynamically deposited and stripped on the Zn surface. As shown in Figure 1g, uncontrollable Zn deposition and even flake‐like Zn dendrites generally occurred on Zn substrate mainly due to nonuniform distribution and sluggish diffusion of Zn ions at the electrode/electrolyte interface. To circumvent the irreversibility issues, t‐KTN protective layer was directly coated on Zn foil by the doctor blading method. Prior to this, as‐prepared t‐KTN crystals were ground into fine powder to obtain a good film‐forming ability (Figure S8, Supporting Information). SEM analysis and corresponding EDX mapping results (Figure S9, Supporting Information) of the t‐KTN‐coated Zn metal (donated as Zn@t‐KTN) foil reveal the porous surface morphology with uniformly‐distributed t‐KTN nanoparticles. Moreover, the t‐KTN layer with a thickness of ≈15 µm is observed to be tightly attached to the Zn foil from the cross‐sectional SEM image (Figure S10, Supporting Information). The contact angle tests show that t‐KTN coated Zn foils exhibit better electrolyte wettability than pure Zn foil (Figure S11, Supporting Information), indicating that porous t‐KTN layer is beneficial to electrolyte penetration and promotes Zn ion transport towards anode surface. Ionic conductivity and Zn ion transference number (*t*_Zn_^2+^) of the t‐KTN layer play a key role in the enhanced performance of Zn anode. As shown in Figure S12, Supporting Information, the ionic conductivity barely changes when varying the thickness from 2 to 50 µm, and the t‐KTN layer with the thickness of 15 µm exhibits an ionic conductivity of about 2.8 mS cm^−1^. The *t*_Zn_^2+^ of the t‐KTN layer was assessed via the DC polarization and electrochemical impedance spectroscopy (EIS) measurements (Figure S13, Supporting Information). The *t*_Zn_^2+^ of the Zn@t‐KTN electrode is calculated to be 0.65, whereas this value is only 0.35 for the bare Zn foil. The high ionic conductivity and transference number indicate the t‐KTN layer can reduce the Zn ion concentration gradient and accelerate the Zn ion migration at the ferroelectric/electrode interface.

The ferroelectric‐domain mediated mechanism of the t‐KTN layer to dynamically regulate the deposition behavior of Zn metal is illustrated in Figure 1h. Owing to the spontaneous polarization of t‐KTN, plenty of cations and anions from the electrolyte environment will be strongly chemisorbed onto the ferroelectric surface with the counter‐charges respectively, depending on the direction of ferroelectric polarization. Zn ions are driven to adsorb on the negatively charged surface of t‐KTN and deposit at the ferroelectric/Zn interface through the electrostatic interaction between cations and negatively charged surface with downward polarization states. In view of the periodic distribution of ferroelectric polarized domains, the adjacent/neighboring domains with upward polarization states attract anions, thus preventing the electrodeposition of Zn. With the synergy of the ferroelectric polarizations and domain configurations, there is a formation of interlamellar patterns composed of electrodeposited Zn in the initial stage of Zn deposition. The well‐aligned Zn interlamellar arrays with low tortuosity can not only facilitate Zn ion transport and reduce ion concentration gradient, but also modulate selective deposition within interlamellar arrays.

To gain insight into the migration of Zn ions in the ferroelectric surface, we performed density functional theory (DFT) calculations. The simulated model is based on the t‐KTN crystal and the potential energy surface along the migration pathway of Zn ion is depicted in Figure S14, Supporting Information. t‐KTN is a solid‐solution perovskite crystal with KTaO_3_ and KNbO_3_. During the diffusion process, the Zn ion is able to pass through the remaining interspace consisting of octahedral TaO_6_ and NbO_6_ in the framework of t‐KTN crystal. The migration pathway of Zn ion is displayed in the insets. The estimated migration energy barriers of Zn ions imply the high Zn‐ion diffusion rate through the t‐KTN layer.

**Figure 2:**
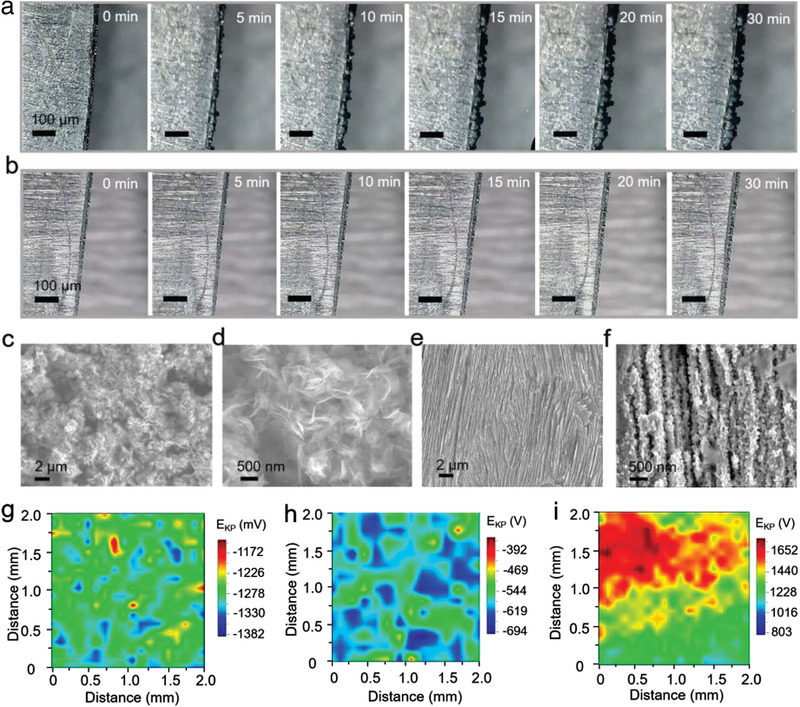
In situ optical microscopy, SEM investigation, and SKP analysis of Zn@t‐KTN electrode. a,b) In situ optical observations of Zn deposition behavior on (a) bare Zn and (b) Zn@t‐KTN electrodes at 5mA cm^−2^. c–f) SEM images of Zn deposition morphology on (c, d) bare Zn and (e, f) Zn@t‐KTN electrodes at 1 mA cm^−2^ (0.5 mAh cm^−2^). g–i) Volta potential maps of (g) fresh Zn, (h) cycled Zn@t‐KTN, and (i) cycled Zn electrodes via SKP, the t‐KTN layer was removed before the test.

To visually verify the effect of t‐KTN layer on Zn dendrite growth, a symmetric cell configuration was assembled to in situ observe Zn deposition behavior via optical microscopy. Zn electrodeposition was conducted under a high current density of 10 mA cm^−2^ in a transparent home‐made cell. As expected, uneven Zn electrodeposits with tiny protrusions randomly appear on the surface of bare Zn electrode as early as 10 min of Zn plating (**Figure** **2**a). With the increase of plating time, these tiny protrusions are inclined to grow into disordered Zn dendrites, and the thickness of deposited Zn layer gradually becomes much larger, resulting in poor Zn reversibility and short circuits of battery. For the cell using Zn@t‐KTN electrodes, a compact and flat deposition morphology without protrusions can be observed during the whole plating process (Figure 2b), leading to a homogeneous electric field distribution and high Zn reversibility. It is demonstrated that uncontrolled and dendritic Zn growth can be addressed by achieving ferroelectric domain‐mediated Zn deposition behavior at the ferroelectric‐electrolyte interface.

The protective layers on the cycled Zn anodes were carefully removed by methyl‐2‐pyrrolidinone (NMP) to dissolve polyvinylidene difluoride (PVDF) binder before SEM characterization. Under a deposition capacity of 1 mAh cm^−2^, the bare Zn anode displays a rugged morphology with sharp and disordered Zn flakes (Figure 2c, d), which might pierce the separator during cycling. In contrast, the Zn@t‐KTN presents a dense and uniform morphology with well‐aligned interlamellar structured Zn deposits (Figure 2e). Specifically, it can be clearly found that numerous regular Zn crystals are clung to the Zn lamellar walls through inward ion‐transport paths (Figure 2f). The flake‐like morphology on the cycled Zn electrode was almost fully sustained before and after the NMP treatment (Figure S15, Supporting Information), demonstrating that non‐uniform morphology of the deposited Zn and the generated by‐products cannot be affected. The variations in morphology should be ascribed to the homogenous current density distribution enabled by the well‐aligned lamellar structure with low tortuosity, resulting in selective deposition within interlamellar arrays (Figures S16 and S17, Supporting Information).

To figure out the mechanism of Zn ion diffusion, chronoamperometry (CA) test was performed on symmetric cells under a constant overpotential of −150 mV. For bare Zn electrode, a sharp increase in the current density within 50 s indicates a rampant 2D diffusion process associated with uneven nucleation (Figure S18, Supporting Information). The increase in the surface area of Zn anode is mainly due to the uneven Zn deposition. In contrast, the current density of Zn@t‐KTN electrode remains almost unchanged after only 10 s, reflecting a constant and uniform 3D diffusion process after initial nucleation and short 2D diffusion. The much lower current density for the Zn@t‐KTN anode demonstrates that Zn deposition is very even and dense without the increase of effective electrode area. In consideration of the interplay between ferroelectric dipoles and adsorbed Zn ions, the unique domain configuration with periodically distributed polarization direction can effectively restrict random diffusion of Zn ions and regulate the concentration distribution of Zn ions at the ferroelectric‐electrolyte interface. As a consequence, uniform Zn deposition prefers to occur in the negatively charged regions with upward polarization states. In addition, hydrogen evolution reaction (HER) overpotential obtained from linear sweep voltammetry (LSV) curves confirms the protective effect of t‐KTN layer on suppressing HER in aqueous electrolyte (Figure S19, Supporting Information).

To investigate the protective effect of t‐KTN layer on Zn electrode, scanning Kelvin probe (SKP) technology was implemented to measure the Volta potential distribution on electrode surface. Figure 2g–i shows the Volta distribution on the bare Zn, and Zn@t‐KTN electrodes before and after Zn plating/stripping process. Before cycling, the Volta potential distribution on bare Zn electrode fluctuates in a narrow range from −1172 to −1382 mV and becomes relatively dispersed/heterogeneous (Figure 2g and Figure S20a, Supporting Information). After 10 cycles of Zn plating/stripping, the Volta potential distribution on Zn@t‐KTN electrodes is obviously shifted positively to the value with a narrow range from −700 to −396 mV (Figure 2h and Figure S20b, Supporting Information), indicating that t‐KTN protective layer can prevent aqueous electrolyte from corroding Zn anode, suppress dendrite growth and alleviate the formation of non‐uniform passivation layer on the electrode. As a comparison, the Volta potential of bare Zn electrodes is distributed uniformly and the average values concentrate around 1350 mV (Figure 2i). This result implies that the uneven and ionic‐insulating passivation layer generated by side reactions can increase the work function of Zn electrode surface.

**Figure 3:**
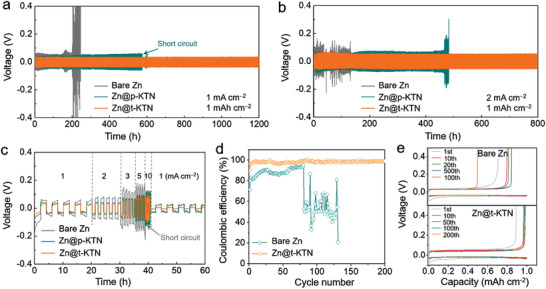
Electrochemical performance and Zn plating/stripping behaviors of Zn@t‐KTN electrode. Galvanostatic Zn plating/stripping cycling in symmetric cells of bare Zn, Zn@p‐KTN and Zn@t‐KTN electrodes at the current densities of a) 1 mA cm^−2^ with a capacity of 1mAh cm^−2^, and b) 2 mA cm^−2^ with a capacity of 1mAh cm^−2^. c) Rate performance of symmetric cells of bare Zn, Zn@p‐KTN, and Zn@t‐KTN electrodes from 1 to 10 mA cm^−2^ with a capacity of 1 mAh cm^−2^. d) CEs of the Zn plating/stripping on asymmetric cells with t‐KTN‐coated stainless steel (t‐KTN@SS) electrode and bare stainless steel (SS) electrodes at 2 mA cm^−2^ with the capacity limited to 1 mAh cm^−2^. e) Voltage profiles of the t‐KTN@SS and SS electrodes at 2 mA cm^−2^ with the capacity limited to 1 mAh cm^−2^.

To evaluate the electrochemical stability of Zn@t‐KTN electrode, galvanostatic cycling test of symmetric cells was performed under different current densities. The symmetric cell with Zn@t‐KTN electrode exhibits a stable voltage profile with a low voltage hysteresis of 30 mV for 1200 h under a current density of 1 mA cm^−2^ and areal capacity of 1 mAh cm^−2^ (**Figure** **3**a). In contrast, a sharp increase in polarization voltage appears on bare Zn electrode after 200 h of cycling at the same test condition, mainly resulting from the accumulation of by‐products and competitive hydrogen evolution on the anode surface. Besides, paraelectric KTN (p‐KTN) crystal without ferroelectric and piezoelectric properties was also used as an intermediate layer (Figures S21–S25, Supporting Information). As expected, Zn@p‐KTN electrode displays a larger voltage hysteresis and a shorter cycle lifespan (582 h) than the Zn@t‐KTN electrode, indicating the ineffectiveness of p‐KTN layer due to the rampant growth of Zn dendrites. More importantly, under a higher current density of 2 mA cm^−2^ and areal capacity of 1 mAh cm^−2^, Zn@t‐KTN can still be stably cycled with a constant voltage hysteresis of 40 mV for over 800 h (Figure 3b). However, rapid battery failure and fluctuated voltage plateaus simultaneously occur on bare Zn electrode after only 30 h, whereas the Zn@p‐KTN electrode suffers from a sudden voltage increase after 460 h, which could be attributed to the severe side reactions and formation of Zn dendrites. Even under the harsh cycling conditions of 10 mA cm^−2^ and 5 mAh cm^−2^, the symmetric cell using Zn@t‐KTN electrodes also exhibited excellent cycling performance with a steady polarization voltage for 300 h (Figure S26, Supporting Information). From the Nyquist plots of the bare Zn symmetric cell in Figure S27a, Supporting Information, due to the formation of the by‐products and the “dead” Zn on the anode surface, the transfer resistance of the Zn||Zn cell increases quickly after 100 cycles. In contrast, the semicircle diameters in the high‐frequency region almost maintained unchanged after 100 cycles because of the ferroelectric domain‐mediated Zn deposition on the Zn@t‐KTN (Figure S27b, Supporting Information). The electrochemical performance of Zn@p‐KTN is competitive to recently reported modified Zn metal anode with various protective layers (Table S1, Supporting Information). Figure 3c compares the voltage profiles of bare Zn, Zn@p‐KTN, and Zn@t‐KTN at various current densities from 1 to 10 mA cm^−2^. With the increase of current density, the bare Zn electrode shows gradually increased polarization voltages and experiences a sudden voltage drop at a higher current density of 10 mA cm^−2^, indicating an internal short circuit of battery induced by uncontrollable Zn dendrite growth. Compared with bare Zn and the Zn@p‐KTN, the Zn@t‐KTN symmetric cell exhibits substantially lower voltage hysteresis at all current densities, especially at high current densities. These results demonstrate the feasibility of ferroelectric domain‐mediated Zn deposition strategy for Zn metal anode.

The reversibility of Zn plating/stripping process was further evaluated by the CE of the asymmetric cell. As illustrated in Figure 3d, the CE of Zn//SS asymmetric cell steadily increases to 96% in the initial stage and fluctuates rapidly after 75 cycles at 2 mA cm^−2^ with a capacity of 1 mAh cm^−2^. This cell failure might result from the dendrite‐induced localized short‐circuiting. By contrast, the asymmetric cell with the t‐KTN@SS electrode maintains an average CE of about 97.6% after a few cycles, which indicates the excellent reversibility of Zn plating/stripping after the short activation and stabilizing process of t‐KTN@SS electrode. Besides, the voltage hysteresis of t‐KTN@SS electrode is smaller than those of bare SS electrode, indicating the enhanced Zn transfer kinetics at the ferroelectric‐electrolyte interface (Figure 3e).

The electrochemical corrosion of the electrode was quantitatively analyzed by the linear polarization measurements in 1 m ZnSO_4_ aqueous electrolyte. The linear polarization curves (Figure S28, Supporting Information) show that the corrosion potential (*E*_corr_) of the Zn@t‐KTN electrode (−1.014 V) is much higher than that of Zn foil (−1.022 V), indicating effective suppression of HER and dissolved oxygen‐induced passivation in the presence of t‐KTN layer. In addition, the corrosion current density of Zn@t‐KTN electrode is significantly reduced from 0.86 to 0.28 mA cm^−2^ as compared with that of Zn foil, revealing the lower rate of self‐corrosion reactions. Thus, the improved electrochemical performance of the Zn@t‐KTN electrode can be attributed to its good corrosion‐resistant performance.

**Figure 4:**
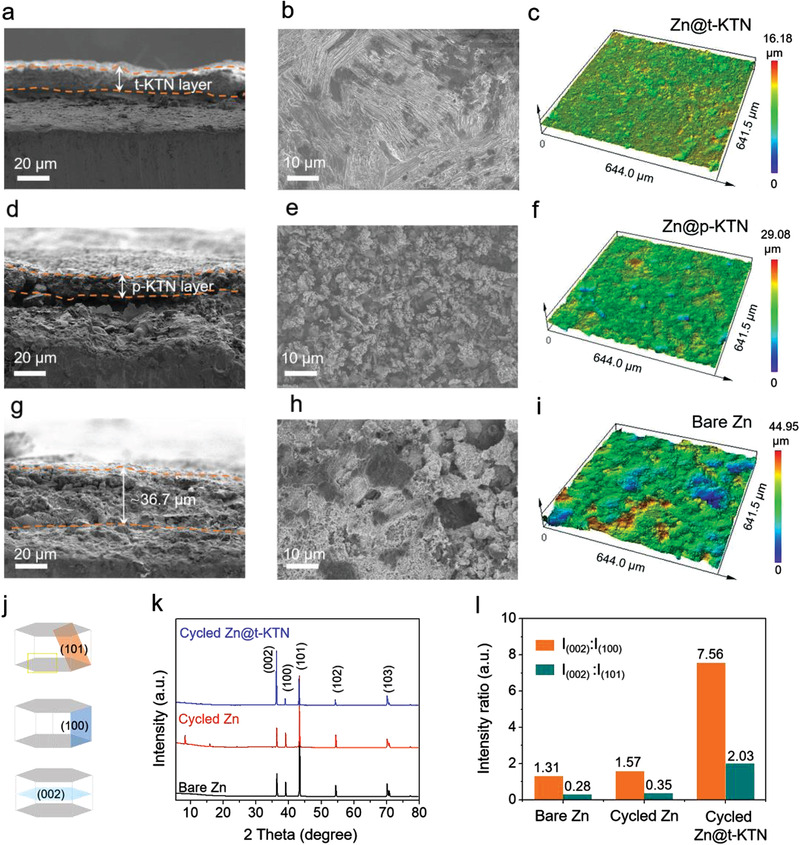
Morphology evolution of the Zn@t‐KTN anodes. a–i) Side‐view and top‐view SEM and CLSM optical images of the (a–c) cycled Zn@t‐KTN, (d–f) Zn@p‐KTN, and (g–i) bare Zn electrodes after 100 cycles. j) Schematic illustrations of the spatial relationship of the (101), (100), and (002) crystalline planes of hexagonal Zn. k) XRD patterns for bare Zn, cycled Zn, and cycled Zn@t‐KTN electrodes. l) The average peak intensity ratios of (002) plane to (100) and (101) crystal planes.

SEM images of Zn anodes extracted from symmetric cells further verify the protective effect of the t‐KTN layer. As shown in **Figure** **4**a, the t‐KTN layer is tightly and evenly attached to the surface of Zn metal anodes. To further verify the adhesion stability of t‐KTN layer, the cycled Zn@t‐KTN electrode extracted from full cell was characterized by SEM. The result demonstrated that t‐KTN layer is still attached to Zn foil (Figure S29, Supporting Information). After removing the KTN layer, the exposed Zn electrode surface still maintains a smooth surface without dendrite growth after repeated Zn stripping/plating (Figure 4b). The confocal laser scanning microscope (CLSM) (Figure 4c) shows surface height difference is only 16.18 µm. These results demonstrate the t‐KTN layer can regulate the Zn‐ions diffusion and suppress dendrite growth at the ferroelectric/electrode interface. For Zn@p‐KTN electrode, vertical Zn dendrites appear on the surface of uncovered Zn anode and the p‐KTN layer has poor contact with the Zn foil due to the generation of obvious void space at the interface (Figure 4d, e). Meanwhile, the Zn deposition morphology is much rougher with an average height difference of 29.08 um (Figure 4f). More seriously, a porous and dendritic layer with a depth up to 36.7 um is observed on the surface of cycled bare Zn electrode, and the value of height difference reaches 44.95 um (Figure 4g–i), suggesting the poor reversibility, severe side reactions and uncontrolled Zn deposition occurring on bare Zn anode. From the different deposition morphologies, it is believed that the ferroelectric domain configuration plays an important role in adjusting Zn stripping/plating behavior and the unique domain structure of t‐KTN crystals with periodically distributed polarization directions can successfully confine the Zn deposition in the specific region with upward polarization states (negatively charged surface).

To clarify the crystal structure of Zn electrodeposits, XRD patterns collected on the cycled Zn and Zn@t‐KTN electrodes are compared in Figure 4j–l. The preferential deposition orientation of metallic Zn can be determined by the intensity ratios of the diffraction peaks.^[^ ^33^, ^47^ ^]^ We calculated the intensity ratios of different diffraction peaks using diffraction peaks of (002) plane as the reference. As summarized in Figure 4l, the cycled Zn electrode shows a similar peak intensity ratio of (002) to (101) as compared to the bare Zn, which illustrates the dominant deposition orientation of Zn crystal along (101) planes. The deposited Zn in the (101) direction with a high angle of 30–70° to the substrate would induce disordered crystal phase growth, and eventually transform into Zn dendrites. Remarkably, the intensity ratio of the (002) to (101) for cycled Zn@t‐KTN electrode is much higher than those of bare Zn and cycled Zn electrodes. The dominant (002) plane present on the cycled Zn@t‐KTN electrode confirms that t‐KTN layer can promote the growth of Zn along the preferential direction approximately parallel to the anode surface. In addition to the Zn crystal peaks, several new diffraction peaks appear on the cycled Zn electrode, which are attributed to irreversible byproduct Zn_4_(OH)_6_SO_4_ *x*H_2_O (PDF#44‐0673). Conversely, there are almost no peaks of these Zn ion‐insulating impurities observed on cycled Zn@t‐KTN electrode, indicating a highly reversible and stable Zn plating process.

**Figure 5:**
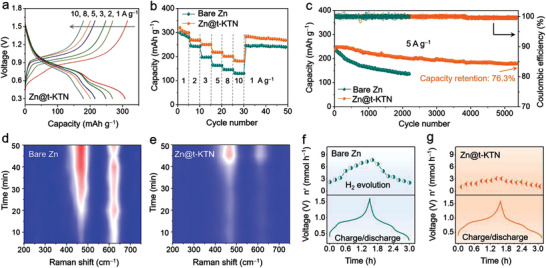
Electrochemical performances of Zn@t‐KTN//NVO full cell. a) Galvanostatic charge/discharge profiles of Zn@t‐KTN//NVO full cell at various current densities. b) Rate capability of bare Zn//NVO and Zn@t‐KTN//NVO full cells at various current densities. c) Long‐term cycling performance of bare Zn//NVO and Zn@t‐KTN//NVO at a rate of 5 A g^−1^. In situ Raman profiles of the d) bare Zn//NVO and e) Zn@t‐KTN//NVO cells. In situ monitoring of hydrogen evolution rate on f) bare Zn//NVO and g) Zn@t‐KTN//NVO full cell during the first cycle.

To assess the feasibility and effectiveness of Zn@t‐KTN anode in full cells, NaV_3_O_8_ 1.5H_2_O (NVO) cathodes were coupled with bare Zn and Zn@t‐KTN anodes. Detailed characterizations of the NVO cathode are shown in Figure S30, Supporting Information. **Figure** **5**a, b compares the rate capability of the Zn//NVO batteries based on bare Zn and Zn@t‐KTN anodes at various current densities. The specific capacities of Zn//NVO batteries with bare Zn and Zn@t‐KTN anodes are comparable at the low current densities, whereas the battery with Zn@t‐KTN anodes gradually outperforms that of bare Zn anode with the increasing current densities. When the current density is switched back to 1 A g^−1^, the battery with Zn@t‐KTN anode delivers a high capacity of 284 mAh g^−1^, which is higher than that with bare Zn anode (252 mAh g^−2^), manifesting the improved rate performance of the Zn@t‐KTN anode. The long‐term stability of Zn//NVO batteries based on bare Zn and Zn@t‐KTN anodes was also examined at a high current density of 5 A g^−1^. As displayed in Figure 5c, the specific capacity of Zn//NVO battery rapidly decays from 242 to 127 mAh g^−1^ after 2200 cycles with capacity retention of only 52.4%. Benefitting from the effective t‐KTN layer, the Zn@t‐KTN//NVO battery presents a stable cycle life with a high capacity‐retention of 76.3% (the initial capacity of 237 mAh g^−1^) after 5500 cycles, which is superior to the Zn//NVO battery.

We then carried out in situ Raman spectroscopy to monitor the side reactions on Zn anode. For the bare Zn foil in ZnSO_4_ electrolyte, the existence of inactive Zn(OH)2− 4 species can be assigned to an obvious peak at 465 cm^−1^ during the deposition process, which suggests a high concentration of OH^−^ ions in local regions of Zn electrode. Locally increased pH would inevitably cause water‐induced Zn metal corrosion and even disordered dendrite formation. In comparison, the peak intensity of Zn(OH)2− 4 species in the Zn@t‐KTN//NVO cell is obviously weaker than that of bare Zn//NVO, indicating that the corrosion reaction of Zn@t‐KTN electrode is significantly suppressed during the whole deposition process. The hydrogen gas evolution during Zn plating/stripping in full cells was investigated by a battery‐gas chromatography‐mass spectrometry (GC‐MS) system (Figure S31, Supporting Information). The full cells were cycled with a current density of 0.2 A g^−1^. As shown in Figure 5f, the hydrogen evolution rate rapidly increases upon charging in bare Zn//NVO full cell. Conversely, negligible release of hydrogen gas is detected in Zn@t‐KTN//NVO full cell (Figure 5g), which suggests the hydrogen evolution can be suppressed during the entire charge process with the protection of t‐KTN layer. The surface morphologies of Zn anodes retrieved from full cells after 1000 cycles at 5 A g^−1^ are shown in Figure S32, Supporting Information. For the cycled bare Zn anode, an uneven Zn deposition with disordered dendrites is found on the surface. In sharp contrast, Zn@t‐KTN electrode presents a flat and compact surface with uniform Zn deposition. These results indicate that the suppression of side reaction and dendrite growth is mainly due to regulation of cation distribution and anion concentration gradient at the interface from ferroelectric polarization of the t‐KTN layer.

## 3 Conclusion

In summary, we report a novel ferroelectric domain‐mediated strategy to manipulate the Zn plating behavior and achieve controllable Zn growth orientation by coating a ferroelectric tetragonal KTN layer on the surface of Zn foil. The ferroelectric domain of t‐KTN crystal exhibits the periodic distribution of upward and downward polarizations, which can synergistically regulate cation distribution and anion concentration via the interplay between ferroelectric dipoles and adsorbed ions. With the synergistic effect of the ferroelectric polarization and domain configurations, the formation of well‐aligned Zn interlamellar arrays with low tortuosity in the initial stage of Zn deposition could enable selective deposition within the interlamellar arrays. As a result, Zn@t‐KTN anode exhibits superior Zn stripping/plating behavior with low voltage hysteresis and long lifespan of 1200 h at 1 mA cm^−2^ in symmetric cells. When paired with the NVO cathode, the full cell delivers an ultralong cycling stability with 76.3% capacity retention after 5500 cycles at a high current density of 5 A g^−1^. We believe that this novel ferroelectric domain‐mediated metal deposition strategy can also be extended to other metal anodes for high‐energy rechargeable batteries.

## 4 Experimental Section

### Synthesis of t‐KTN Single Crystals

KTa_1‐x_Nb_x_O_3_ (KTN) single crystals were grown via an improved top‐seeded solution growth (TSSG) method. The raw materials of potassium carbonate (K_2_CO_3_, 99.99%), tantalum pentoxide (Ta_2_O_5_, 99.99%), and niobium pentoxide (Nb_2_O_5_, 99.99%) powders were weighed according to the phase diagram. The raw material was heated at 200 °C for 4 h in advance for removing the water absorbed on the surface. The mole ratio of K_2_CO_3_, Ta_2_O_5_, and Nb_2_O_5_ was 1.04: 0.54: 0.46. Based on the KTN phase diagram, a three‐order fitting of the solidus curve reveals the relationship between growth temperature and the Nb component of KTN crystal as:

(1) $$T=-102.4x^{3}+383x^{2}-596.3x+1352$$

where *T* is the growing temperature of KTN, *x* is Nb component of the growing crystal. Therefore, the gradient of *x* can be determined by the changes of growing temperatures.

### Synthesis of Modified Zn Electrodes

The Zn@t‐KTN composite materials were prepared by a simple blade coating method according to previous reports with minor modifications. t‐KTN crystals were ground into tiny particles, and then was mixed with polyvinylidene fluoride (PVDF) binder at a weight ratio of 9:1 into N‐methyl pyrrolidinone (NMP) solvent. The above slurry was pasted onto polished Zn foil with a thickness of 50 µm. After vacuum drying at room temperature for 24 h, Zn@t‐KTN electrode was obtained. Besides, Zn@p‐KTN was prepared by the same method, except for replacing t‐KTN with p‐KTN powder.

### Synthesis of NaV_3_O_8_ 1.5H_2_O Cathodes

NaV_3_O_8_ 1.5H_2_O nanobelts were prepared through a facile liquid‐solid stirring method. 1.0 g of commercial V_2_O_5_ powder was added to 25 mL flask containing 15 mL of 2 m NaCl aqueous solution. The mixture solution was stirred at 30 °C for 96 h. Then, the suspension was centrifuged at 7000 rpm for 10 min. Finally, the product was obtained by vacuum‐drying. The NVO cathode was prepared by mixing 70 wt.% NVO powder, 20 wt.% acetylene black carbon, and 10 wt.% PVDF binder in NMP solvent, and then the slurry was cast onto carbon paper by a doctor‐blade method. After drying at 60 °C overnight in a vacuum oven, the NVO cathode was obtained and was punched into disks with a diameter of 12 mm. The specific capacity of the NVO cathode was calculated based on the weight of the active materials. The areal mass loading for the NVO electrodes was about 3.5 mg cm^−2^.

### Material Characterizations

The coin cells were disassembled to obtain the cycled anodes. The protective layers on the cycled Zn anodes were removed by methyl‐2‐pyrrolidinone (NMP) to dissolve PVDF binder before characterization. The phase structure of t‐KTN crystal was determined by X‐ray diffractometry (XRD, Bruker D8) with Cu K*α* radiation (*λ* = 1.5406 Å). The morphologies of the obtained t‐KTN and Zn anodes were examined using SEM (ZEISS Supra 40) equipped with an energy dispersive X‐ray (EDX) spectrometer. TEM, high angle annular dark‐field scanning transmission electron microscopy (HAADF‐STEM) were performed using FEI Titan G2 80–200 microscope operating at 200 kV. The polarization microscope images were observed by a polarizing microscope (Axioskop40, Zeiss). The vertical piezo‐response force microscopy (V‐PFM) images were obtained by using a commercial microscope (Cypher ES, Asylum Research) with conductive Pt/Ir‐coating probes (EFM, Nanoworld). The applied voltage was 500 mV. Contact angle measurements were conducted by using a KRUSS DSA100 machine. The CLSM images of samples have been collected on Keyence VK‐X200 microscope.

### Electrochemical Measurements

The full cells, symmetric cells, and half cells were assembled as coin‐type cells (CR2025) to measure the electrochemical performances. Symmetric cells were composed of two pure Zn (Zn@p‐KTN or Zn@t‐KTN) electrodes, which were separated by a glass fiber membrane in 1 m zinc sulfate (ZnSO_4_) electrolyte. All the Zn foils with a thickness of 50 µm were applied. Half‐cells were assembled by the bare Zn foil (Zn@p‐KTN or Zn@t‐KTN) as the counter electrode and the reference electrode, and Ti foil as the working electrode. The full cells were assembled with as‐prepared NVO electrode as the cathode, glass fiber as the separator, and Zn@t‐KTN (or bare Zn) as the anode in CR2025 coin cells. The electrolyte consists of 1 m ZnSO_4_ and 0.1 m Na_2_SO_4_ for the Zn||NVO full cell. CA measurements were performed on a Gamry Reference 600+ electrochemical workstation at −150 mV overpotential for Zn metal batteries. Exchange current densities were measured by fitting Tafel plots of Zn||Zn symmetric coin cells at a scan rate of 5 mV s^−1^ in a voltage range of −0.8 to −1.1 V. Cyclic voltammetry (CV) and EIS data were measured by the same instruments for the symmetric cells. EIS was performed in the frequency range of 10^5^–10^−1^ Hz with a potential amplitude of 10 mV. The electrochemical performance of full cells was tested in a voltage range of 0.3–1.6 V. An in situ optical microscope was used to observe the morphology evolution of bare Zn and Zn@t‐KTN electrodes in symmetric cells during Zn plating process. The bare Zn and Zn@t‐KTN electrodes were covered with glass plates to construct a homemade transparent optical cell in order to investigate the interfacial stability.

### DFT Calculation Method

The DFT calculations were performed using the Perdew–Burke–Ernzerhof (PBE) functional together with plane‐wave projected augmented wave (PAW) method as implemented in Vienna Ab initio Simulation Package (VASP). To investigate the migration of Zn ions within the t‐KTN crystal, a 2×2×1 supercell model of t‐KTN was created. The kinetic energy cutoff for the plane‐wave basis set was set to be 500 eV. A 3×6×6 Gamma‐centered k‐point grids in the first Brillouin zone were generated using the Monkhorst–Pack sampling scheme. Long‐range vdW interactions have been taken into account with Grimme's scheme. The t‐KTN supercell was fully optimized with convergence criteria of 1×10^−5^ eV for total energy and 1×10^−2^ eV Å^−1^ for the root‐mean‐square residual force, respectively.

### In Situ Raman Measurements

In situ Raman electrochemical cell with a quartz window was used for Raman spectroscopy analysis. The Raman spectra were acquired using a Bruker Raman microscope with an excitation length of 532 nm. Each spectrum was acquired for 30s, and the interval time was 10 s over a potential range of 0.3–1.6 V. Total spectra were obtained at the first cycle.

### Scanning Kelvin Probe (SKP) Measurements

SKP measures the work function difference between a substrate and a reference probe to acquire high‐spatial mapping of the surface potential distribution. The scanning area was 2 × 2mm^2^.

Because of the differences in elemental composition of the tip and the sample, there was a contact potential difference (*V*_CPD_) that exists between the two materials as defined by

(2) $$V_{\mathrm{CPD}}=\left(\omega_{s}-\omega_{t}\right)/q$$

where *V*_CPD_ represents Volta potential of tested point, *ω*_s_ and *ω*_t_ represent tested point and probe escape works, and *q* represents electric quantity of a charge. *V*_CPD_ referred to the Volta potential of tested point. The Volta potential maps of cycled bare Zn and Zn@t‐KTN electrodes were recorded before removing the t‐KTN layer.

## Conflict of Interest

The authors declare no conflict of interest.

## Supporting information

Supporting Information

## Data Availability

The data that support the findings of this study are available from the corresponding author upon reasonable request.
