# Supplementary material for: Unveiling the Synergistic Effect of Ferroelectric Polarization and Domain Configuration for Reversible Zinc Metal Anodes
Source: Adv Sci (Weinh). 2022 Mar 10;9(14):2105980. doi: 10.1002/advs.202105980 (PMC9108597; doi:10.1002/advs.202105980)
Supplement: Supplementary file 1 — Supporting Information [file ADVS-9-2105980-s001.pdf]

ADVANCED SCIENCE
Open Access

Supporting Information

for *Adv. Sci.*, DOI 10.1002/advs.202105980

Unveiling the Synergistic Effect of Ferroelectric Polarization and Domain Configuration for Reversible Zinc Metal Anodes

Tao Chen, Fei Huang, Yinan Wang, Yi Yang, Hao Tian* and Jun Min Xue**

WILEY-VCH

© 2022 Wiley-VCH GmbH

Supporting Information

Unveiling the synergistic effect of ferroelectric polarization and domain configuration for reversible zinc metal anodes

Tao Chen, Fei Huang, Yinan Wang, Yi Yang, Hao Tian, Jun Min Xue

Dr. T. Chen
School of Chemistry and Chemical Engineering
Nanjing University of Science and Technology
Nanjing 210094, China
E-mail: chen_tao20@sina.cn

Dr. T. Chen, Y. Yang, Prof. J. M. Xue
Department of Materials Science and Engineering
National University of Singapore
117575, Singapore
E-mail: msexuejm@nus.edu.sg

Dr. Y. Wang
School of Mathematical Science
Peking University
Beijing 100871, China

Dr. F. Huang, Prof. H. Tian
School of Physics
Harbin Institute of Technology
Harbin, 150001, China
E-mail: tianhao@hit.edu.cn

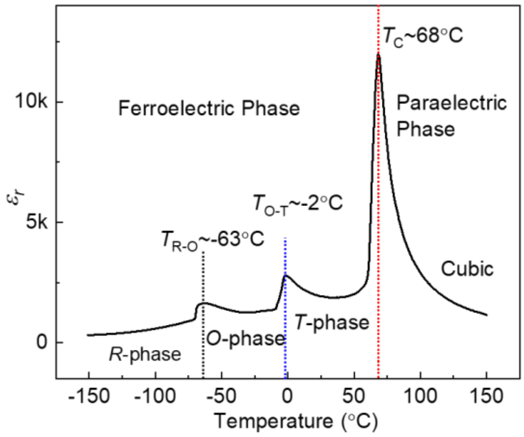

Figure S1. The curves of ε_r versus temperature for t-KTN crystal.

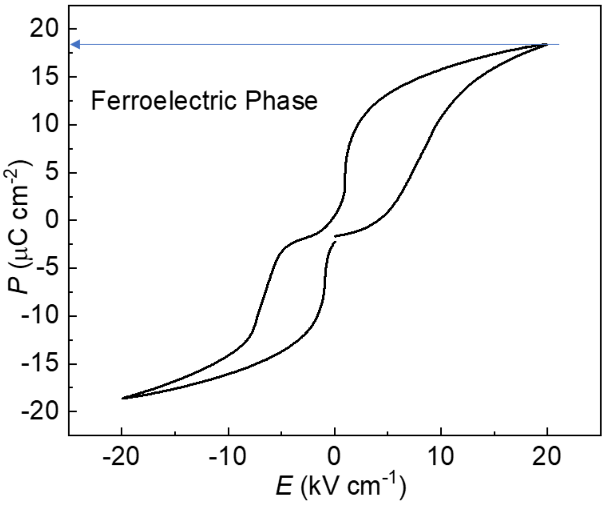

Figure S2. P-E hysteresis loop of as-grown t-KTN crystal

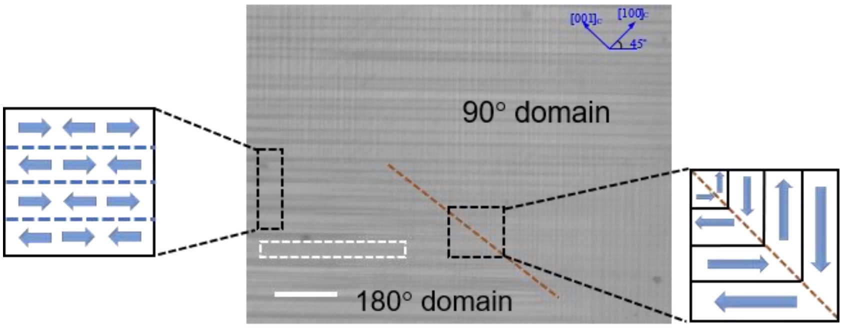

Figure S3. Polarizing light microscope image of the t-KTN crystal. The scale bar is 2 μm. The white dotted line represents the 180° domain wall, and the red dotted line shows the 90° domain wall.

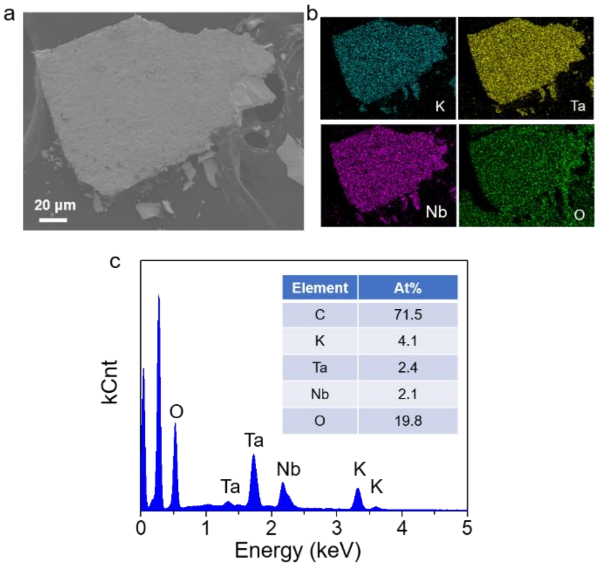

Figure S4. SEM image, corresponding elemental mapping images, and EDS analysis of t-KTN crystal.

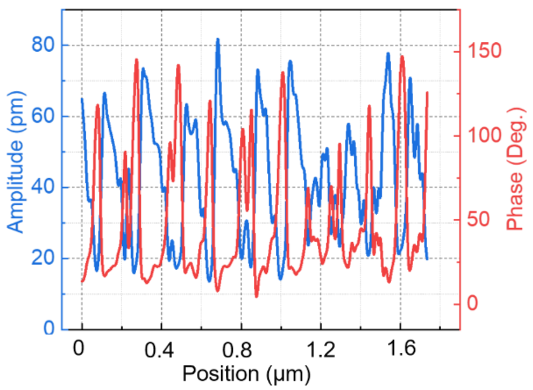

Figure S5. The details of amplitude and phase derived from white dotted lines in Figure 1e, f, showing that the differences of the amplitude and phase are still 180°.

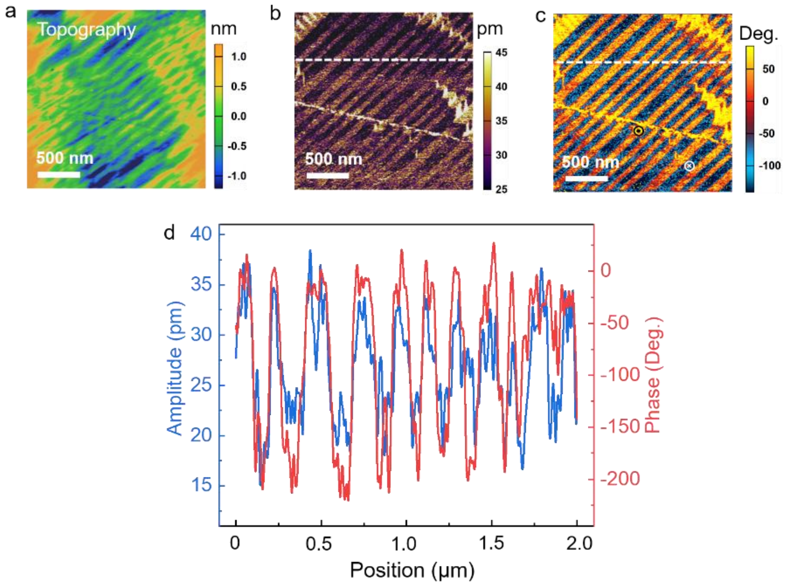

Figure S6. The out-of-plane piezoresponse force microscope images of 90° domain regions in KTN crystal for surface topography (a), amplitude (b), and PFM phase (c). (d) The details of amplitude and phase derived from white dotted lines in Figure S5b, c, showing that the differences of the amplitude and phase are 90°.

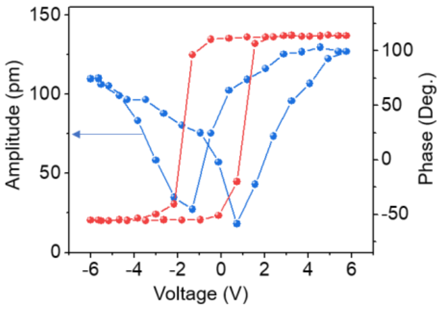

Figure S7. Curves of piezo-responsive amplitudes and phases of the t-KTN crystals.

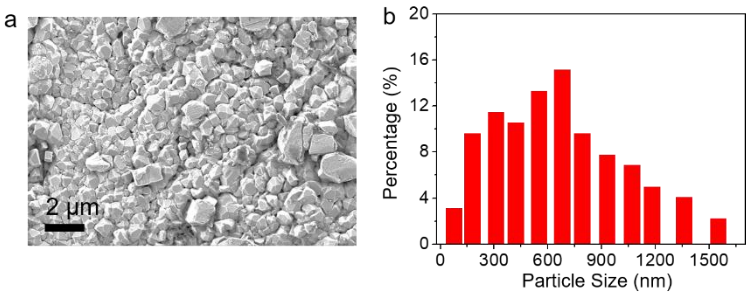

Figure S8. SEM image of grinded t-KTN powders and corresponding particle size distribution.

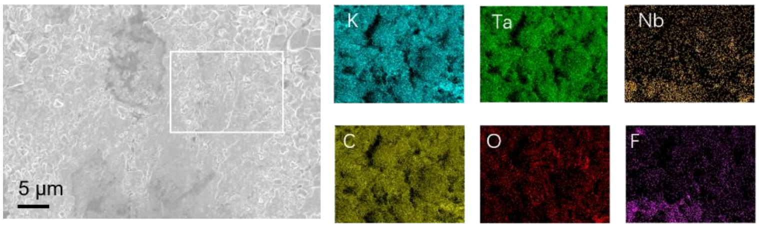

Figure S9. SEM image of t-KTN layer and corresponding elemental mapping images of K, Ta, Nb, C, O, F elements.

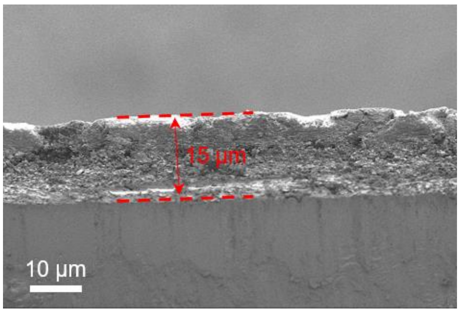

Figure S10. Cross-sectional SEM image of Zn@t-KTN electrode with a thickness of 15 μm.

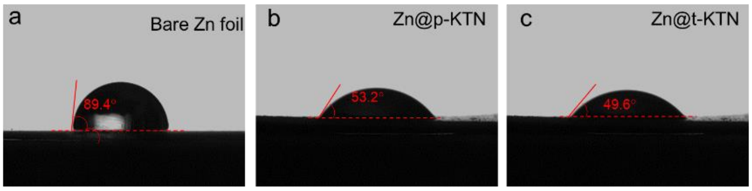

Figure S11. Contact angles of $ZnSO_4$ electrolyte (5μL) on (a) bare Zn foil , (b) Zn@p-KTN and (c) Zn@t-KTN.

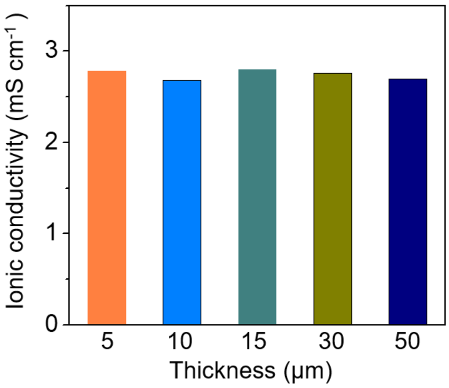

Figure S12. Ionic conductivity of t-KTN layer with various thickness of 5, 10, 15, 30, and 50 μm.

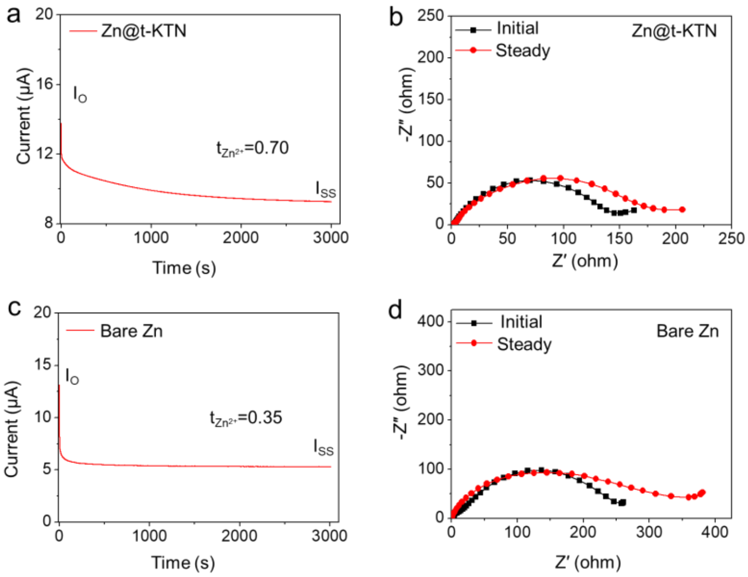

Figure S13. The chronoamperometry profiles of symmetric cells with Zn@t-KTN (a) and bare Zn (c) electrodes under a polarization voltage of 10 mV. The corresponding electrochemical impedance spectra (EIS) before and after polarization of symmetric cells with Zn@t-KTN (b) and bare Zn (d) electrodes.

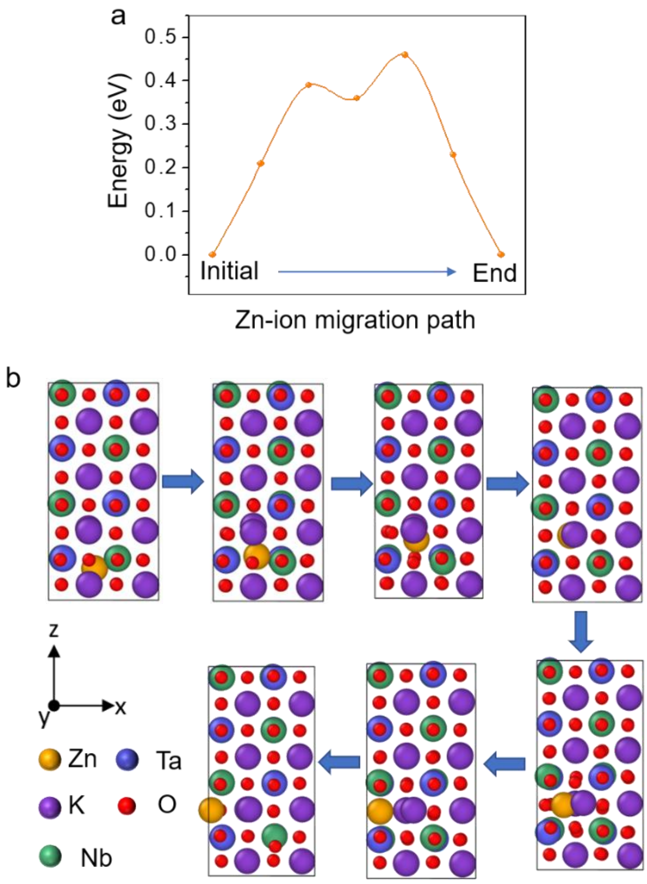

Figure S14. The energy barrier profiles (a) and migration pathway (b) of Zn^{2+} ion schematically showing the diffusion occurs through the following step: Zn atom pass through the remaining interspace consisting of octahedral TaO_6 and NbO_6 in the framework of t-KTN crystal.

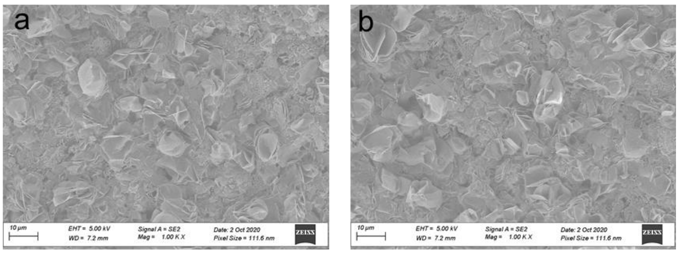

Figure S15. SEM images of the cycled Zn anode before (a) and after (b) washing with NMP solvent.

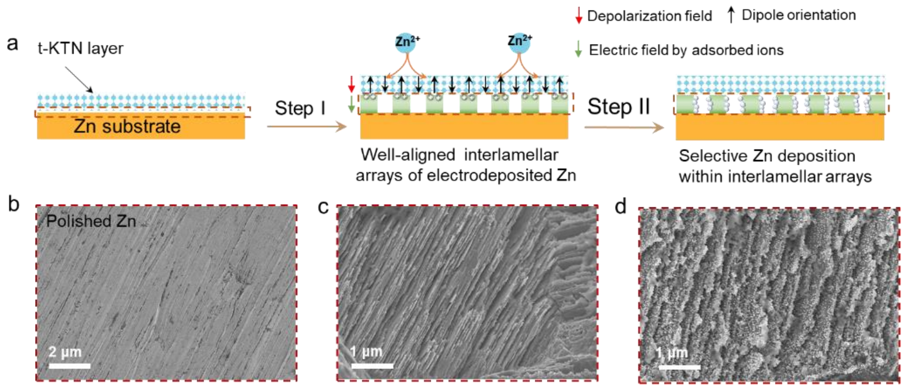

Figure S16. The plating pattern of Zn metal on Zn@t-KTN electrode. a) Schematic illustration of Zn deposition on Zn@t-KTN, in which the protective layer of Zn@t-KTN has been removed. b–c) Top-view SEM images of polished Zn (b) and Zn metal deposited on Zn@t-KTN with a plating capacity of 0.5 mAh cm^{-2} (c), 1 mAh cm^{-2} (d) respectively.

The charged ferroelectric surfaces can manipulate the transfer kinetics of Zn ions and the concentration distribution of anions via the interplay between ferroelectric dipoles and adsorbed ions (**Figure S16a**). In the initial deposition process, the well-aligned interlamellar arrays composed of electrodeposited Zn are formed (**Figure S16b,c**). In the subsequent process, Zn ions are more preferentially electrochemically reduced and uniformly deposited on the Zn lamellar walls after traveling the shortest vertical channels (**Figure S16d**).

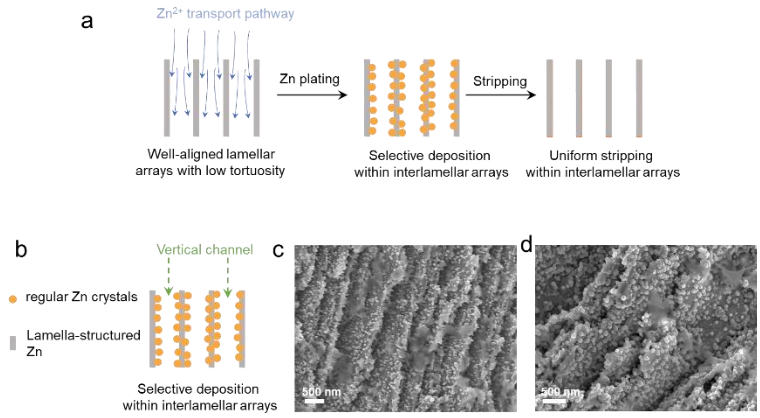

Figure S17. (a) Schematic of the effect of well-aligned lamellar structure with low tortuous during deposition. Because of the low tortuosity of the well-aligned lamellar structure, numerous regular Zn crystals were clung to the Zn lamellar walls through inward ion-transport paths. (b) Schematics of Zn deposition in interlamellar arrays. (c and d) SEM images of Zn deposition morphology on Zn@t-KTN electrodes after 10 cycles (c) and 40 cycles (d) at 1 mA cm^{-2}.

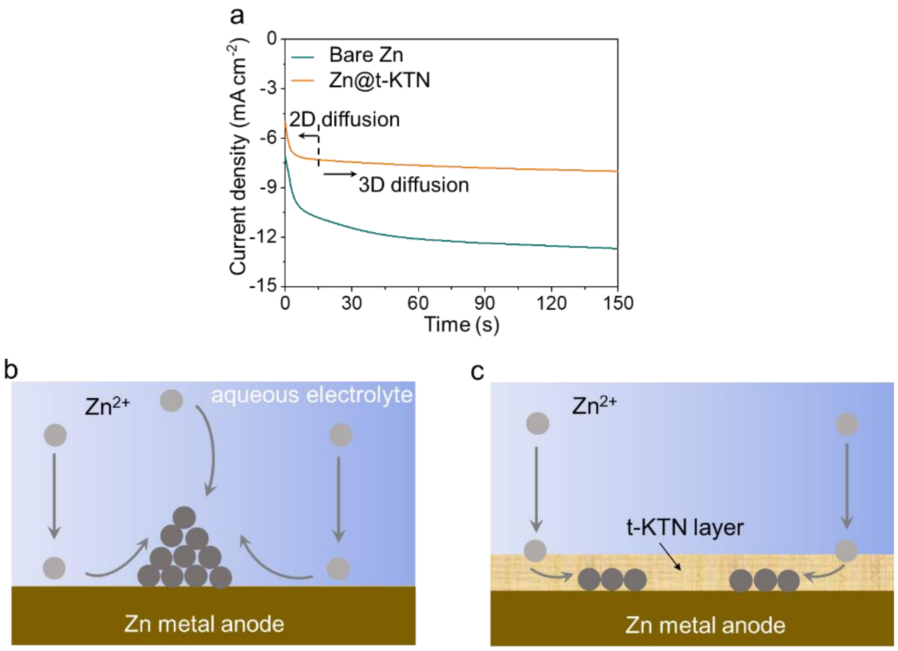

Figure S18. (a) Chronoamperograms of bare zinc foils and Zn@t-KTN electrodes under -0.15 V within 150 s. Schematically diagrams of the Zn^{2+} diffusion and deposition processes on bare Zn (b) and Zn@t-KTN (c) electrodes, showing that the 2D diffusion is constrained on Zn@t-KTN electrodes.

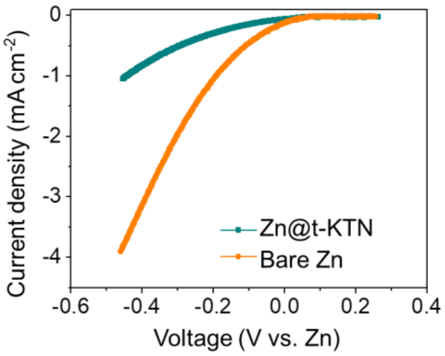

Figure S19. Hydrogen evolution reaction (HER) of Ti||Zn coin cells with bare Zn and Zn@t-KTN electrode in 1 M Zn_2SO_4 electrolyte.

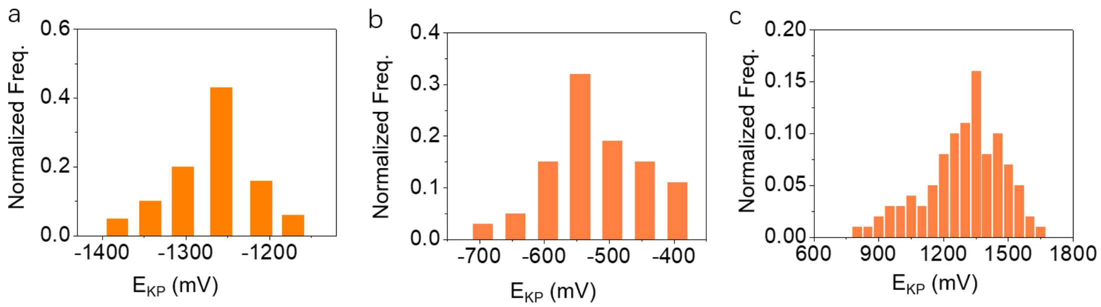

Figure S20. The data statistics of Volta potential maps of (a) fresh Zn, (b) cycled Zn@t-KTN, and (c) cycled Zn electrodes.

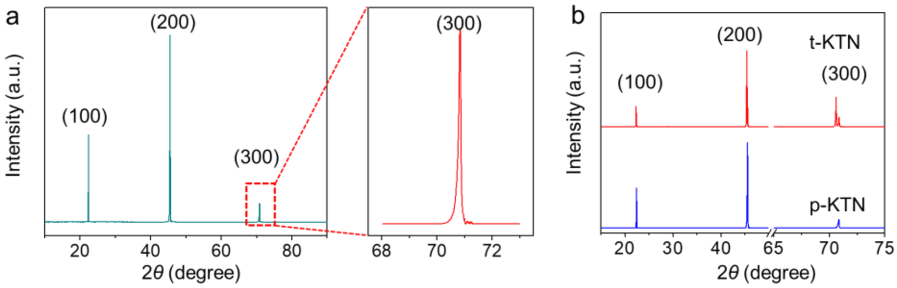

Figure S21. (a) X-ray diffraction pattern of the paraelectric KTN (p-KTN) crystal. (b) X-ray diffraction patterns of the t-KTN and p-KTN.

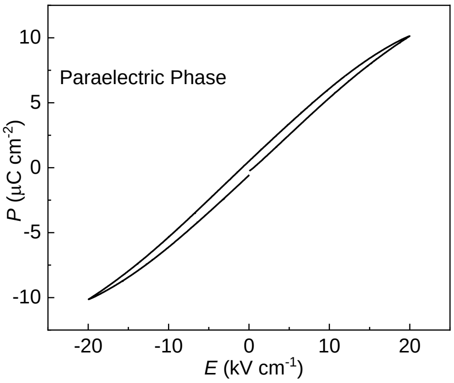

Figure S22. P-E hysteresis loop of as-grown p-KTN crystal.

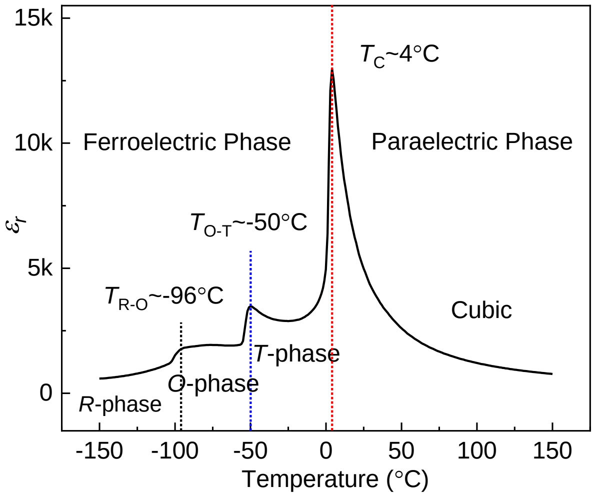

Figure S23. The curves of ε_r versus temperature for p-KTN crystal.

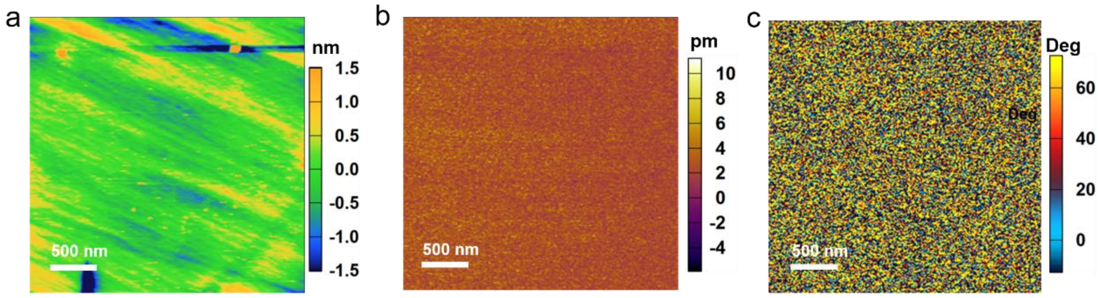

Figure S24. The out-of-plane piezo-response force microscope images of p-KTN crystal for **a** surface topography, **b** amplitude, and **c** PFM phase.

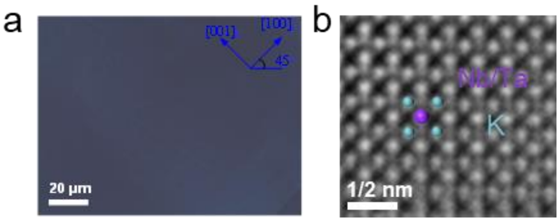

Figure S25. (a) Polarizing light microscope image of the p-KTN crystal, showing no domain structure. (b) Fast fourier transform showing the displacement of the B-site ions at the atomic scale in p-KTN.

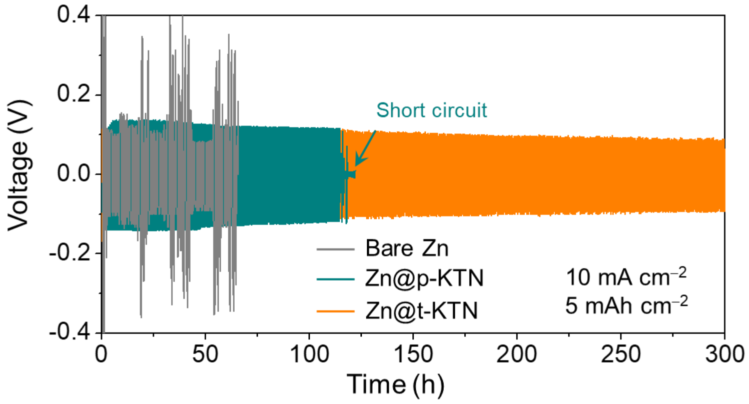

Figure S26. Galvanostatic Zn plating/stripping cycling in symmetric cells of bare Zn, Zn@p-KTN and Zn@t-KTN electrodes at the current densities of 10 mA cm^{-2} with a capacity of 5 mAh cm^{-2}.

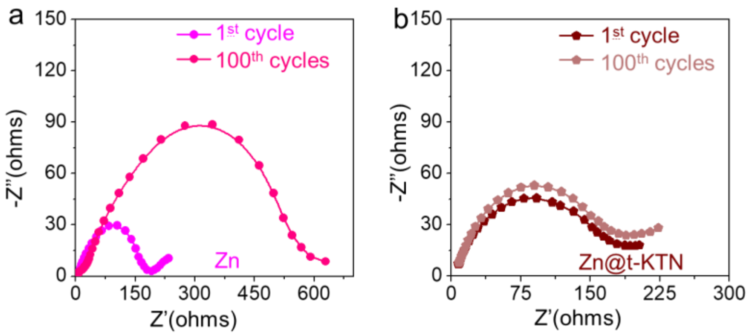

Figure S27. Comparisons of EIS spectra for bare Zn (a), Zn@t-KTN (b) symmetric batteries after the 1st and 100th cycles.

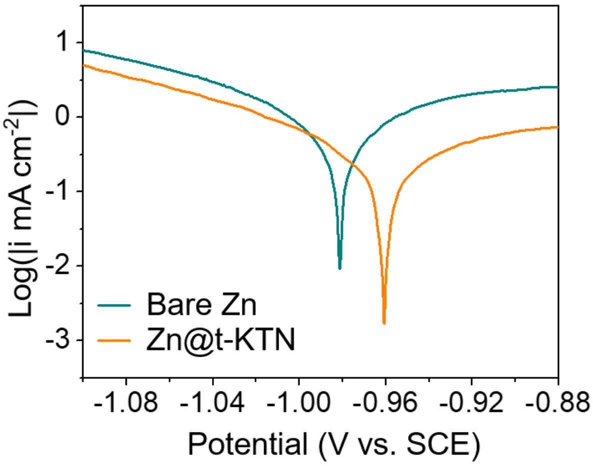

Figure S28. Linear polarization curves of bare Zn and Zn@t-KTN electrodes at a scan rate of 1 mV s^{-1} using three-electrode system.

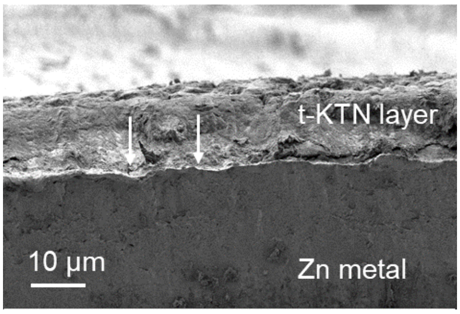

Figure S29. Side-view SEM image of the cycled Zn@t-KTN in full-cell configuration.

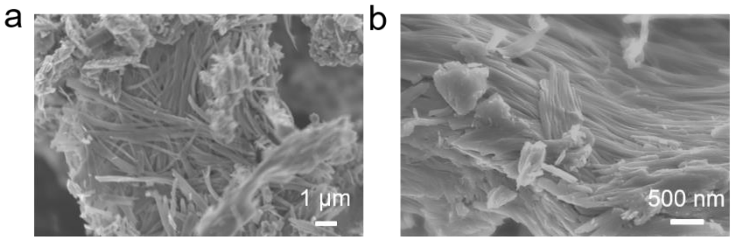

Figure 30. SEM images of $NaV_3O_8{\cdot}1.5H_2O$ nanobelts.

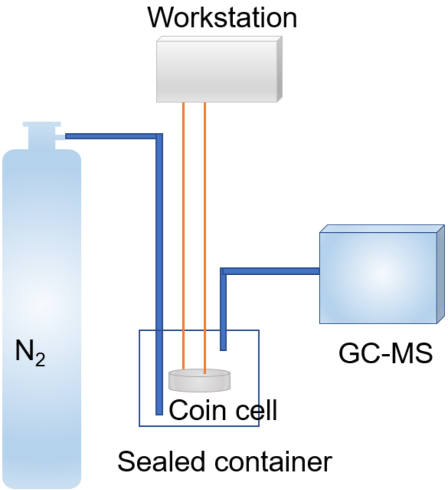

Figure 31. Schematic illustration of the home-built system for in situ detection of hydrogen gas evolution using battery-gas chromatography-mass spectrometry.

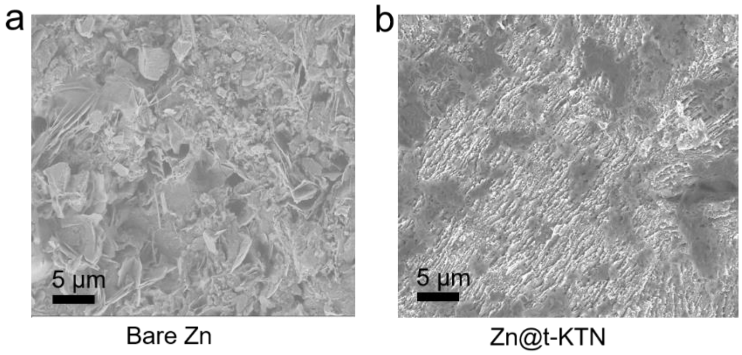

Figure 32. SEM images of bare Zn (a) and Zn@t-KTN (b) electrodes after 2000 cycles at a rate of 5 A g^{-1} (t-KTN layer was removed before SEM observation).

Table S1. Summary of electrochemical performance of Zn metal anodes coated the various protective layer.

Protective layer	Current density (mA cm^{-2})	Cycling capacity (mAh cm^{-2})	Cycle life (h)	Ref.
Nanoporous $CaCO_3$	0.25	0.05	836	1
Nafion-Zn-X	1<br>2	0.5<br>0.5	1000<br>1000	2
Faceted TiO_2	1<br>2	1<br>2	460<br>280	3
Mxene ($Ti_3C_2T_x$)	0.2	0.2	800	4
$BiTiO_3$	1	1	4000	5
ZnF_2	0.5<br>1	1<br>1	500<br>800	6
$NaTi_2(PO_4)_3$	1	1	250	7
montmorillonite	1	0.25	1000	8
MOF coating	1<br>2	1<br>2	400<br>400	9
Nitrogen-doped graphene	1	1	1200	10
Sn-crystal texture	0.5<br>1<br>2	1<br>1<br>1	500<br>500<br>320	11
Si_3N_4/Polyacrylonitrile	0.25	0.25	800	12
COFs	1<br>2	1<br>1	420<br>250	13
t-KTN	1<br>2	1<br>2	1200<br>800	This work

Supplementary References

1. Kang, L. T. et al. Nanoporous $CaCO_3$ coatings enabled uniform Zn stripping/plating for long-life zinc rechargeable aqueous batteries. *Adv. Energy Mater.* **8**, 1801090 (2018).
2. Cui, Y. et al. An interface bridged organic-inorganic layer suppressing dendrite and side reactions for ultra-long life aqueous Zn metal anodes. *Angew. Chem., Int. Ed.* **59**, 16594 –16601 (2020).
3. Zhang, Q. et al. Revealing the role of crystal orientation of protective layers for stable zinc anode. *Nat. Commun.* **11**, 3961 (2020).
4. Zhang, N. et al. Direct self-assembly of MXene on Zn anodes for dendrite-free aqueous zinc-ion batteries. *Angew. Chem. Int. Ed.* **60**, 2861–2865 (2021).
5. Zou, P. et al. Ultrahigh-rate and long-life Zinc–metal anodes enabled by self-accelerated cation migration. *Adv. Energy Mater.* **11**, 2100982 (2021).
6. Yang, Y. et al. Synergistic Manipulation of Zn^{2+} Ion Flux and Desolvation E□ect Enabled by Anodic Growth of a 3D ZnF_2 Matrix for Long-Lifespan and Dendrite-Free Zn Metal Anodes. *Adv. Mater.* **33**, 2007388 (2021).
7. Liu, M. et al. $NaTi_2(PO_4)_3$ Solid-State Electrolyte Protection Layer on Zn Metal Anode for Superior Long-Life Aqueous Zinc-Ion Batteries. *Adv. Funct. Mater.* **30**, 2004885 (2020).
8. Yan, H., Li, S., Nan, Y., Yang, S. & Li. B. Ultrafast zinc–ion–conductor interface toward high-rate and stable zinc metal batteries. *Adv. Energy Mater.* **11**, 2100186 (2021).
9. Yuksel, R., Buyukcakir, O., K. Seong, W. & Ruo□, R. S. Metal-Organic Framework Integrated Anodes for Aqueous Zinc-Ion Batteries. *Adv. Energy Mater.* **10**, 1904215 (2020)
10. Zhou, J. et al. Ultrathin Surface Coating of Nitrogen-Doped Graphene Enables Stable Zinc Anodes for Aqueous Zinc-Ion Batteries. *Adv. Mater.* **33**, 2101649 (2021).
11. Li, S. et al. Toward planar and dendrite-free Zn electrodepositions by regulating Sn-crystal textured surface. *Adv. Mater.* **33**, 2008424 (2021).
12. Zhou, S. et al. Anti-Corrosive and Zn-Ion-Regulating Composite Interlayer Enabling Long-Life Zn Metal Anodes. *Adv. Funct. Mater.* 2104361 (2021)
13. Park, J. H. et al. Self-assembling films of covalent organic frameworks enable long-term, efficient cycling of zinc-ion batteries. *Adv. Mater.* **33**, 2101726 (2021).
